# The association between longitudinal patterns of adverse childhood experiences, and self-harm and depression in adolescence and early adulthood: findings from the Avon longitudinal study of parents and children

**DOI:** 10.1007/s00787-025-02781-y

**Published:** 2025-06-06

**Authors:** Bushra Farooq, Abigail E. Russell, Kate Allen, Laura D. Howe, Becky Mars

**Affiliations:** 1 Centre for Academic Mental Health, Population Health Sciences, University of Bristol Medical School, Bristol, UK; 2 Children and Young People’s Mental Health Research Collaboration, University of Exeter Medical School, Exeter, UK; 3 MRC Integrative Epidemiology Unit, Population Health Sciences, University of Bristol Medical School, Bristol, UK; 4 Centre for Academic Mental Health, Population Health Sciences, National Institute for Health and Care Research, Biomedical Research Centre, University of Bristol Medical School, Bristol, UK

**Keywords:** ALSPAC, Adverse childhood experiences, Self-harm, Depression, Childhood adversity, Latent class analysis, Longitudinal cohort

## Abstract

Adverse childhood experiences (ACEs) elevate the risk of self-harm and depression, which are significant public health concerns. This study examined the association between the longitudinal co-occurrence patterns of ACEs across childhood and adolescence, and self-harm and depression in adolescence and early adulthood. We included 8,859 young people from the Avon Longitudinal Study of Parents and Children (ALSPAC). We examined ten ACEs reported prospectively and retrospectively by parents and children throughout childhood and adolescence, and retrospectively by cohort members in adulthood. Self-harm and depression were self-reported in adolescence (age 16 years) and early adulthood (ages 23 and 24 years). Logistic regression models examined associations between previously derived latent classes of co-occurring ACEs over time, and self-harm and depression. A higher risk of adolescent self-harm was found for each of the latent classes when compared to the *Low ACEs* reference class (*n* = 6,380) (*Mid-childhood and adolescence ACEsn* = 230; adjusted odds ratio (aOR) 1.76, 95% CI 1.11–2.80; *Early childhood abuse and parental mental health problemsn* = 445; aOR 1.85, 95% CI 1.34–2.56; *Persistent parental mental health problemsn* = 861; aOR 1.44, 95% CI 1.07–1.94; *Early and mid-childhood household disharmonyn* = 943; aOR 1.47, 95% CI 1.10–1.97). The association between *Persistent parental mental health problems* and self-harm persisted in early adulthood (aOR 1.57, 95% CI 1.16–2.12). For adolescent depression, associations were found with the *Early childhood abuse and parental mental health problems* (aOR 2.89, 95% CI 2.20–3.79), *Persistent parental mental health problems* (aOR 1.54, 95% CI 1.18–2.01), *and Early and mid-childhood household disharmony* (aOR 1.36, 95% CI 1.03–1.81) classes when compared to the *Low ACEs* class. These associations persisted in early adulthood. *Early childhood abuse and parental mental health problems* (aOR 2.50, 95% CI 1.68–3.73) and *Early and mid-childhood household disharmony* (aOR 1.58, 95% CI 1.05–2.37) were associated with a higher likelihood of co-occurring self-harm and depression in adolescence. However by early adulthood, only *Persistent parental mental health problems* were associated with a higher likelihood of co-occurring self-harm and depression (aOR 2.13, 95% CI 1.47–3.10). ACEs occurring throughout childhood and adolescence are associated with a higher risk of adolescent self-harm and both adolescent and early adulthood depression. In contrast, the class most strongly associated with early adult self-harm was *Persistent parental mental health problems*. Multicomponent interventions and multi-sectoral preventative efforts are needed to address distinct patterns of co-occurring ACEs during childhood and adolescence, to mitigate their short- and long-term impacts on self-harm and depression.

**Supplementary Information:**

The online version contains supplementary material available at 10.1007/s00787-025-02781-y.

## Introduction

Self-harm and depression in young people are major public health concerns worldwide. Depression is one of the most common mental health disorders, affecting approximately 23.4 million children and adolescents under the age of 20 globally, with one-year and lifetime prevalence rates of major depressive disorder estimated at 8% and 19%, respectively [1–3]. Depression is episodic in nature, with recurrence rates ranging from 45 to 48% during adolescence and early adulthood [4, 5]. Adolescent onset is associated with a greater risk of recurrence in adulthood and with more persistent symptoms and poorer long-term outcomes [6]. Self-harm, meanwhile, emerges during early adolescence and peaks in mid-adolescence; it is associated with premature mortality and a range of poor psychosocial outcomes in adulthood [7–9]. The prevalence of self-harm in the past 12 months is estimated to range between 8% and 24% among those aged 12–18 years [10–14]. Self-harm and depression are known to commonly co-occur, with studies reporting almost a half of those with depression engage in self-harm behaviours [15]. Young people may harm themselves to relieve negative affect associated with mental disorders such as depression, and depression has been identified as a risk factor for self-harm with suicidal intent in adolescents [16, 17]. Additionally, the presence of both self-harm and depression is associated with a heightened risk of suicide [18]. In the UK, self-harm and depression are highlighted in the national suicide prevention strategy for England due to their heightened risk for suicide [19]. Understanding the risk factors for these outcomes–both when they occur independently (self-harm alone or depression alone) and together (co-occurring self-harm and depression)– is critical for informing the development of interventions and public health prevention strategies, as both depression and self-harm have significant short and long-term consequences on mental health and well-being [8, 20, 21].

Adverse childhood experiences (ACEs) such as abuse, neglect, and household dysfunction have been linked to increased risk of self-harm and depression [22, 23]. ACEs are common in the population and known to co-occur, as experiencing one increases the risk of experiencing additional ACEs [24]. For example, the co-occurrence of child maltreatment, household substance abuse, and domestic violence has previously been reported [25]. The co-occurrence of ACEs is commonly examined using a cumulative count of the total number of adversities experienced by each individual and used as a predictor for health outcomes [22]. However, this approach can mask important differences in the effects of different ACEs, and the synergistic effects of different combinations of ACEs on outcomes [26].

A growing number of studies have examined ACE co-occurrence using Latent Class Analysis (LCA) [27–29]. LCA is a statistical method used to identify unobservable, or latent, subgroups within a population based on responses to a set of observed categorical indicators [30]. However, existing studies that have used this approach have failed to take account of developmental timing and chronicity of exposure to each ACE [27]. This is an important limitation, as research using other methodologies has demonstrated the significance of timing and chronicity. For instance, one study using a Structured Life Course Modeling Approach found that the accumulation of maternal depression, rather than exposure during specific developmental periods, explained the most variability in depressive symptoms in early adulthood [31]. While this study focused on maternal depression, it highlights a broader issue relevant to ACE research, the need to consider not only the co-occurrence of adversities but also their timing and persistence in relation to subsequent offspring mental health outcomes. Only one previous study examined the clustering of maltreatment considering chronicity of exposure and developmental timing, and found high probabilities of physical, emotional, and sexual abuse occurring both in childhood and adolescence [32]. However, other common ACEs such as parental mental health problems, substance abuse, separation and divorce that co-occur with maltreatment were not examined. Nonetheless, these findings highlight the importance of considering the chronicity and developmental timing of ACEs.

Research on how the co-occurrence of ACEs in childhood and adolescence contribute to the risk of self-harm and depression is sparse. Despite the high co-occurrence of self-harm and depression, no study to our knowledge has examined both self-harm and depression in relation to ACEs within the same sample. This represents a critical gap in the literature, as understanding how ACEs contribute to both outcomes simultaneously could provide insights into shared and distinct mechanisms underlying these mental health outcomes. One study examined the association between patterns of ACE co-occurrence and depression, and found child maltreatment, characterised by high probabilities of physical neglect, physical abuse, and emotional abuse, was associated with increased likelihood of depressive symptoms in adulthood, compared to those experiencing “low adversity” [33]. Similarly, another study found latent classes characterised by high probability of child maltreatment had the most potent impact on depressive symptoms compared those experiencing “low adversity” [34]. In one of the few studies that focussed on self-harm, Xiao et al. found a class characterised by multiple forms of co-occurring adversities was most strongly associated with a higher risk of self-harm [35]. However, the cross-sectional design of this study means that the direction of the relationship between ACEs and self-harm cannot be established. Furthermore, the retrospective reporting of ACEs by participants introduces the potential for recall bias.

To date, no previous study has examined the association between the longitudinal patterns of ACE co-occurrence in childhood and adolescence, considering their developmental timing, and their associations with later self-harm and depression. Existing studies on co-occurring ACEs are often cross-sectional, group childhood and adolescence together, and fail to explore their association with distal mental health outcomes. Furthermore, many studies rely on selective samples (e.g., clinical populations or school students), and only examine a limited number of ACEs, most of which are retrospectively reported [36, 37]. As a result, a gap exists in understanding how the timing and co-occurrence of ACEs in childhood and adolescence affect short and long-term mental health outcomes such as self-harm and depression. It is particularly crucial to explore these associations across both adolescence and early adulthood, as self-harm, despite a decline in prevalence after adolescence, can persist into adulthood, and adolescent-onset depression is a strong predictor of recurrence in later life [38]. Therefore, there is a need for research that specifically examines whether the adverse outcomes linked to ACEs continue beyond adolescence and into early adulthood. To address these gaps, this study aimed to examine associations between the longitudinal patterns of ACE co-occurrence with self-harm and depression in adolescence and early adulthood using data from a prospective longitudinal cohort study of parents and children followed up from pregnancy to adulthood in the UK.

## Methods

### Study population

This population-based study used data from the Avon Longitudinal Study of Parents and Children (ALSPAC). ALSPAC is a longitudinal birth cohort study based in the South West of England, UK. Pregnant women resident in Avon, UK with expected dates of delivery from 1st April 1991 to 31st December 1992 were invited to take part in the study [39, 40]. The initial number of pregnancies enrolled was 14,541. Of these initial pregnancies, there was a total of 14,676 foetuses, resulting in 14,062 live births and 13,988 children who were alive at 1 year of age. Further details are available in supplement (Material S1). ALSPAC contains detailed data on family, socioeconomic, and demographic factors before birth, as well as exposure to adversity, and mental health outcomes measured over multiple time-points in childhood, adolescence, and adulthood. The data is controlled by ALSPAC and is not publicly available, access can be requested via the study website (http://www.bristol.ac.uk/alspac/researchers/access/).

## Measures

### Adverse childhood experiences and derivation of latent classes

Prospective and retrospective measures of different types of ACEs were taken throughout childhood, adolescence, and adulthood, resulting in 348 questionnaire items for the period from birth to 16 years (Supplementary Table S1). Retrospective measures taken in adulthood at age 22 years covered exposure to ACEs before the age of 16 years. Exposure to ten ACEs occurring during three developmental periods were examined: early childhood (birth to 5 years); middle childhood (6 to 10 years); and adolescence (11 to 16 years). ACEs included physical abuse, emotional abuse, emotional neglect, sexual abuse, bullying, domestic violence, substance abuse, parental mental health problems, parent conviction, and divorce/separation. A dichotomous (yes/no) variable indicating exposure to each of the ten types of ACEs were derived for each developmental period. An ACE was assumed present if it was reported by the mother, her partner, or the study child (Supplementary Material S2).

**Fig. 1:**
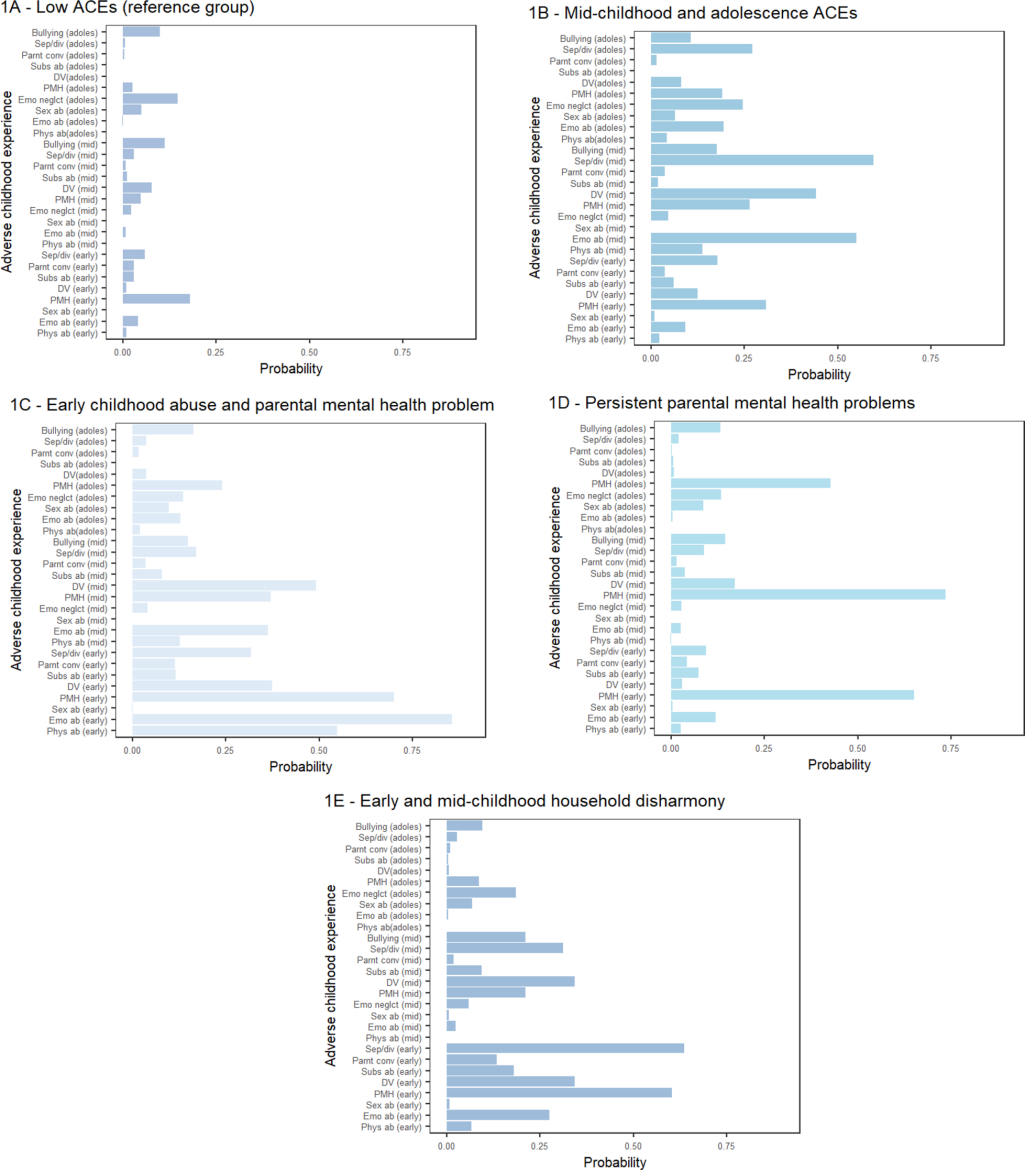
Adverse childhood experience probabilities by latent class

We previously conducted a LCA to examine the longitudinal co-occurrence patterns of ACEs in Mplus (version 8.9), the results are published elsewhere [41] and are available in Supplementary Material S4. Based on model fit, a log-likelihood ratio test, interpretability, plausibility of the class solution, and class size, we selected a 5-class solution as our final model (model fit indices are reported in Supplementary Table S2). The item probabilities for each ACE by latent class are reported in Supplementary Table S3 and shown in Fig. 1. The 5-class solution was labelled:

1. *Low ACEs* (*n* = 6,380, 72.0%), characterised by a low probability of exposure to any type of ACE.
2. *Early and mid-childhood household disharmony* (*n* = 943, 10·6%), characterised by exposure to multiple ACEs related to household disharmony, such as parental separation/divorce, domestic violence, substance abuse, parental mental health problems, and parent conviction, occurring primarily in the early and mid-childhood period.
3. *Persistent parental mental health problems* (*n* = 861, 9·7%), characterised by a high probability of exposure to parental mental health problems at all three developmental periods, with a low probability of other ACEs.
4. *Early childhood abuse and parental mental health problems* (*n* = 445, 5·0%), characterised by a high probability of physical and emotional abuse, and parental mental health problems occurring in early childhood, and domestic violence in mid-childhood.
5. *Mid-childhood and adolescence ACEs* (*n* = 230, 2·6%), characterised by a high probability of ACEs such as emotional abuse and neglect, domestic violence, and parental separation/divorce occurring during mid-childhood and adolescence, with a low probability of ACEs in the early childhood period.

### Self-harm

Self-harm was defined as any act of intentional self-poisoning or self-injury regardless of suicidal intent. Past-year self-harm was assessed using postal questionnaires at ages 16 and 24 years (Supplementary Material S3). Cohort members were asked whether they hurt themselves on purpose in any way and the last time they hurt themselves on purpose. A dichotomous variable indicating the presence or absence of self-harm in the previous 12 months was created.

### Depression

The Short Mood and Feelings Questionnaire (SMFQ) measured recent (within the last 2 weeks) symptoms of depression on a 3-point Likert scale at ages 16 and 23 years. The SMFQ comprises 13 questions measuring affective and cognitive symptoms in children and adolescents [42]. Scores ranged from 0 to 26, a previously established cut-off of ≥ 12 was used to define depression.

### Confounders

Analyses were adjusted for confounders known to be associated with both ACEs and self-harm and depression in the existing literature: parity (collected at 18 weeks gestation), social class (collected at 18 weeks gestation), mother’s age at delivery (collected as baseline sample data), mother’s highest educational qualifications (collected at 32 weeks gestation), and home ownership status (collected at 8 weeks gestation) [16, 43–49]. In sensitivity analyses we additionally adjusted for parental poverty (assessed at 32 weeks gestation) and child’s sex.

### Analyses

A manual bias-adjusted three-step approach was applied to examine associations between the latent classes and self-harm and depression [50]. This is preferred over other methods such as the one-step approach, which estimates the LCA model at the same time as the outcome effect estimates. Such methods result in mis-specified models and could lead to the incorrect number of classes [51]. The bias-adjusted three-step approach produces less biased estimates and does not impact class assignment, the manual implementation (i.e. each step is implemented separately) of this approach is recommended for more complex models [50]. After identifying the optimum class solution (step 1), each person is assigned to one of the derived classes based on their posterior class membership probabilities and then the measurement parameters of the LCA model are fixed (step 2) [50]. The third step involves examining associations between the latent classes and distal outcomes, the logits for the classification probabilities for most likely latent class membership by latent class, estimated during step 2, are incorporated in a command that builds in the uncertainty of class membership [50]. In this study, associations between the ACE latent classes and self-harm and depression were examined using logistic regression. We examined three binary outcomes: (i) self-harm (vs. no self-harm), (ii) depression (vs. no depression), and (iii) co-occurring self-harm and depression (vs. neither/self-harm alone/depression alone). The latent class with low probability of ACEs was used as the reference group.

### Missing data

Each child was required to have at least one ACE measured at each developmental period. This resulted in a sample size of 8,859 children. Full Information Maximum Likelihood (FIML) was then used to handle missing data in the ACE indicators to maximise the use of available data on ACEs.

The confounders had low proportions of missing data (< 10%). Data on past-year self-harm at ages 16 and 24 years was available for 51% and 40% of the sample, respectively, and for depression at ages 16 and 23 years 50% and 38%, respectively. Multiple imputation by chained equations was used to handle missing data among the outcomes and confounders to minimize bias due to selective attrition of families that are more likely to have poor mental health [52]. Despite the high proportion (~ 50%) of missing data in some of the outcomes, multiple imputation has been shown to reduce bias in outcome variables with high proportions of missing data (between 40 and 90%) given that the imputation model is correctly specified and sufficient auxiliary variables are included in the model to make the missing-at-random assumption plausible [53]. Fifty imputed datasets were generated separately by sex, with 30 iterations each, the imputation model included all the variables in the analytic model, and additional auxiliary variables that make the missing-at-random assumption more plausible.

## Results

### Descriptive information

Of the 8,859 children in the study, half were male (50.7%) and the majority were of white ethnic background (96.0%; Table 1). The prevalence of self-harm was higher in adolescence (11.8%) than in early adulthood (9.7%), whereas depression was higher in early adulthood (22.2%) compared to adolescence (16.0%). The prevalence of ACEs by developmental period are presented in Supplementary Table S4, and a comparison of the available-cases, proportion of missing data and variables imputed, and the multiply imputed study sample is presented in Supplementary Table S5. Additionally, a comparison of characteristics by different outcome groupings are presented in Table S6.

**Table 1:** Characteristics of the study sample and prevalence of self-harm and depression

	Pooled proportions from multiply imputed data (*n* = 8,859)
*Outcomes*	
Self-harm (adolescence)	11.8
Self-harm (early adulthood)	9.7
Depression (adolescence)^a^	16.0
Depression (early adulthood)^a^	22.2
Co-occurring self-harm and depression (adolescence)	5.5
Co-occurring self-harm and depression (early adulthood)	5.1
*Characteristics*	
Male	50.7
Ethnicity of child	
Non-white	4.0
White	96.0
Household social class	
Professional	10.4
Managerial and technical	40.2
Skilled non-manual	30.5
Skilled manual	11.4
Partly skilled or unskilled	7.4
Housing	
Owned/mortgaged	81.1
Rented	16.1
Other	2.8
Mother’s qualifications^b^	
CSE	14.9
Vocational	9.1
O level	35.7
A level	25.3
Degree	15.1
Mother’s age at delivery	
Under 20 years	2.3
20–29 years	54.4
30 plus years	43.4
Parity (mean)	0.80

a Measured using the Short Mood and Feelings Questionnaire (SMFQ)bCertificate of Secondary Education (CSE) consists of grades 1 to 5, grade 1 is equivalent to pass at Ordinary level (O level). CSEs and O levels merged into General Certificate of Secondary Education (GCSE) qualifications. Advanced level (A level) qualifications are undertaken by students aged 16 and above, taken over two years leading to qualifications for entrance into higher education. Degree refers to university qualifications

### Longitudinal ACE patterns and self-harm and depression in adolescence and early adulthood

**Fig. 2:**
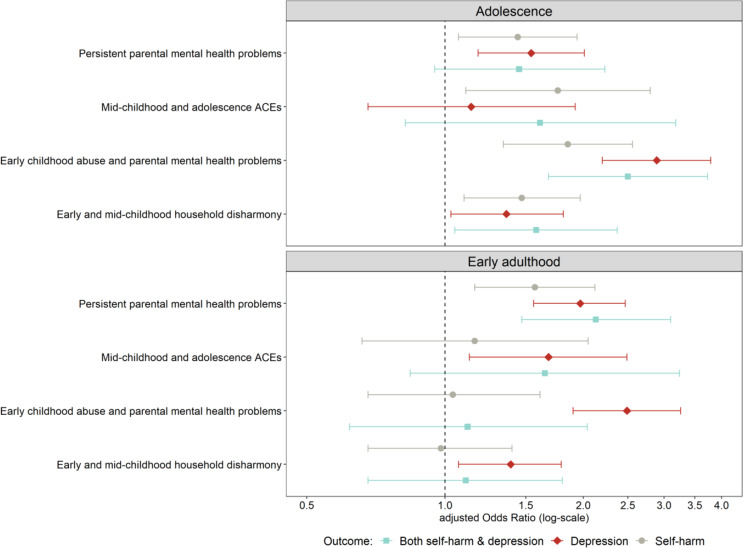
The association between the latent classes (reference group: Low ACEs) and self-harm and depression in adolescence and early adulthood

Unadjusted and adjusted odds ratios (aOR) for the association between latent classes and self-harm and depression in adolescence are presented in Table 2 (adjustment for poverty and sex see Supplementary Table S7). After adjustment for confounders, there was a higher likelihood of self-harm in adolescence among each of the latent classes compared to those in the Low ACEs class (Fig. 2). The aORs ranged from 1.44 (95% CI 1.07–1.94) for *Persistent parental mental health problems* to 1.85 (1.34–2.56) for *Early childhood abuse and parental mental health problems*. Additionally, children exposed to *Early childhood abuse and parental mental health problems* were over two-times more likely to have depression in adolescence than those in the *Low ACEs* class (2.89, 2.20–3.79). Similarly, those in the *Persistent parental mental health problems* (1.54, 1.18–2.01) and the *Early and mid-childhood household disharmony* class (1.36, 1.03–1.81) had a higher likelihood of adolescent depression relative to those in the *Low ACEs* class. No association was found for the *Mid-childhood and adolescence ACEs* class and adolescent depression. For the outcome self-harm with co-occurring depression, associations were only seen among those in the *Early childhood abuse and parental mental health problems* (2.50, 1.68–3.73) and the *Early and mid-childhood household disharmony* classes (1.58, 1.05–2.37).

**Table 2:** Unadjusted and adjusted logistic regression models for the association between latent classes and self-harm and depression in adolescence

	Self-harm OR (95% CI)	Depression OR (95% CI)	Co-occurring self-harm and depression OR (95% CI)	Self-harm OR (95% CI)	Depression OR (95% CI)	Co-occurring self-harm and depression OR (95% CI)
	Model 1 (unadjusted; *n* = 8,859)			Model 2 adjusted for parity, mother’s age at delivery, mother’s qualifications, home ownership, social class (*n* = 8,859).		
Low ACEs (reference group; *n* = 6,380)						
Mid-childhood and adolescence ACEs (*n* = 230)	1.83 (1.15–2.92)	1.17 (0.70–1.95)	1.69 (0.85–3.35)	1.76 (1.11–2.80)	1.14 (0.68–1.92)	1.61 (0.82–3.18)
Early childhood abuse and parental mental health problems (*n* = 445)	1.92 (1.39–2.65)	2.76 (2.10–3.61)	2.54 (1.70–3.79)	1.85 (1.34–2.56)	2.89 (2.20–3.79)	2.50 (1.68–3.73)
Persistent parental mental health problems (*n* = 861)	1.44 (1.07–1.95)	1.53 (1.17-2.00)	1.45 (0.94–2.24)	1.44 (1.07–1.94)	1.54 (1.18–2.01)	1.45 (0.95–2.23)
Early and mid-childhood household disharmony (*n* = 943)	1.80 (1.36–2.39)	1.52 (1.15-2.00)	1.96 (1.33–2.89)	1.47 (1.10–1.97)	1.36 (1.03–1.81)	1.58 (1.05–2.37)

OR = odds ratio

CI = confidence interval

ACEs = adverse childhood experiences

By early adulthood (Table 3), only those in the *Persistent parental mental health problems* class were more likely to report self-harm compared to those in the *Low ACEs* class (1.57, 1.16–2.12). Membership in each of the classes, compared to the *Low ACEs* class, was associated with a higher likelihood of depression in early adulthood; the aORs ranged from 1.39 (1.07–1.79) for *Early and mid-childhood household disharmony* to 2.49 (1.90–3.26) for *Persistent parental mental health problems*. *Persistent parental mental health problems* was additionally associated with a higher likelihood of co-occurring self-harm and depression (2.13, 1.47–3.10).

**Table 3:** Unadjusted and adjusted logistic regression models for the association between latent classes and self-harm and depression in early adulthood

	Self-harm OR (95% CI)	Depression OR (95% CI)	Co-occurring self-harm and depression OR (95% CI)	Self-harm OR (95% CI)	Depression OR (95% CI)	Co-occurring self-harm and depression OR (95% CI)
	Model 1 (unadjusted; *n* = 8,859)			Model 2 adjusted for parity, mother’s age at delivery, mother’s qualifications, home ownership, social class (*n* = 8,859).		
Low ACEs (reference group;; *n* = 6,380)						
Mid-childhood and adolescence ACEs (*n* = 230)	1.20 (0.68–2.11)	1.74 (1.17–2.60)	1.72 (0.88–3.38)	1.16 (0.66–2.05)	1.68 (1.13–2.49)	1.65 (0.84–3.24)
Early childhood abuse and parental mental health problems (*n* = 445)	1.02 (0.66–1.57)	2.39 (1.84–3.11)	1.09 (0.59-2.00)	1.04 (0.68–1.61)	2.49 (1.90–3.26)	1.12 (0.62–2.04)
Persistent parental mental health problems (*n* = 861)	1.56 (1.15–2.10)	1.96 (1.55–2.47)	2.12 (1.46–3.09)	1.57 (1.16–2.12)	1.97 (1.56–2.47)	2.13 (1.47–3.10)
Early and mid-childhood household disharmony (*n* = 943)	1.20 (0.86–1.69)	1.70 (1.33–2.16)	1.46 (0.94–2.27)	0.98 (0.68–1.40)	1.39 (1.07–1.79)	1.11 (0.68–1.80)

OR = odds ratio

CI = confidence interval

ACEs = adverse childhood experiences

## Discussion

To our knowledge, this is the first study to examine the associations of longitudinal ACE patterns, considering their developmental timing of occurrence, and both self-harm and depression in a longitudinal prospective birth cohort in the UK. Compared to individuals with a low probability of ACE exposure, those in classes characterised by Mid-childhood and adolescence ACEs, Early childhood abuse and parental mental health problems, Persistent parental mental health problems, and Early and mid-childhood household disharmony were at a higher risk of self-harm in adolescence. Most of these classes were also associated with a higher risk of depression in both adolescence and early adulthood. For self-harm in early adulthood, only the Persistent parental mental health problems class was associated with a higher risk when compared to the Low ACEs reference class. Similarly, while Early childhood abuse and parental mental health problems and Early and mid-childhood household disharmony were associated with co-occurring self-harm and depression in adolescence, only Persistent parental mental health problems predicted co-occurring self-harm and depression in early adulthood. Among the identified latent classes, Early childhood abuse and parental mental health problems consistently emerged as the highest-risk group, being strongly associated with self-harm, depression, and co-occurring self-harm and depression in adolescence, as well as with an almost threefold higher likelihood of depression by early adulthood.

In a study of ACE co-occurrence by Negriff, higher maltreatment sum scores (i.e. a cumulative score for multiple types of sexual, physical emotional abuse, physical and emotional neglect) were associated with greater depressive symptoms in adolescence, however associations for household dysfunction scores (parental divorce, incarceration, intimate partner violence, substance abuse) were not found [54]. In contrast, we found an association between household disharmony– characterised by indicators of household dysfunction, and depression in both adolescence and early adulthood. This suggests that patterns of co-occurrence and developmental timing of occurrence may be important as opposed to a cumulative count. Our findings are consistent with another study that found latent classes with a high probability of exposure to different types of parental dysfunction and maltreatment, relative to those experiencing few/no ACEs, were associated with self-harm in adolescence [37]. We extend these findings by examining developmental timing of a wide range of ACEs and by exploring associations with self-harm in adulthood. Another study on the clustering of ACEs by Ziobrowski et al. which considered their developmental timing found both men and women exposed to a high probability of emotional abuse in adolescence, and women exposed to childhood physical abuse had higher odds of depressive symptoms in adulthood [32]. Similarly, we found an association between adult depression and the mid-childhood and adolescence ACEs class, (characterised by a high probability of emotional abuse in adolescence, and early childhood abuse and parental mental health problems). However, we did not examine outcomes stratified by sex. Consistent with the present findings, Kim et al. found child maltreatment, characterised by a high probability of physical and emotional abuse, was associated with depression symptoms in adolescence compared to those experiencing few ACEs [33]. Our study additionally found these ACEs co-occurred with parental mental health problems and this class was associated with depression in both adolescence and adulthood.

Exposure to multiple co-occurring ACEs is associated with higher likelihood of self-harm in adolescence, however by early adulthood, associations with self-harm were only observed among those exposed to persistent parental mental health problems. This latent class was additionally associated with depression and co-occurring self-harm and depression in early adulthood. Our findings are consistent with previous research that found children exposed to severe and more chronic parental mental illness have greater distress and depression in adulthood [31, 55]. These findings could also reflect a higher genetic predisposition for mental health problems in offspring [56].

Children exposed to multiple co-occurring ACEs, relative to those with a low probability of ACEs, were more likely to have depression in adolescence and this persisted in early adulthood. The effect estimates were particularly high among those exposed to early childhood abuse and parental mental health problems. Our findings suggest there is an enduring impact of ACEs on mental health over the life course. Chronic recurrent depression in adulthood has been linked to childhood maltreatment [57]. Our findings add to the evidence base and extend previous findings by demonstrating different types of co-occurring ACEs, occurring during childhood and adolescence, are associated with depression both in adolescence and early adulthood.

### Strengths and limitations

The sample included data from a prospective longitudinal cohort study of parents and children followed up at multiple time points from pregnancy through to adulthood. Multiple types of ACEs were included, measured repeatedly throughout childhood and adolescence. This enabled us to comprehensively examine a wide range of ACEs measured both prospectively and retrospectively. We were also able to examine the long-term impact of ACEs on self-harm and depression in adulthood. In addition, we examined the developmental timing of ACEs using advanced statistical methods that address limitations of studies that use a cumulative score to examine ACE co-occurrence, or only consider impacts of individual ACEs in isolation.

There are a number of limitations to consider. First, ACEs were reported prospectively by parents in early childhood, and by children themselves from middle childhood, and retrospectively by young people at age 22 years. It has been shown that retrospective self-reports of maltreatment in childhood are more strongly associated with self-reported mental health problems compared to prospective measures [58]. Second, comparison of child self-reports, and child protection records or reports from multiple informants such as parent’ reports of ACEs show that for child self-reports, perceptions and memories of ACEs may not match what is recorded by others [59]. Nonetheless, we used both prospective and retrospective reports from parents and children, these two data sources have been shown to identify somewhat different groups of the population, and may influence mental health differentially. Third, the ALSPAC sample was recruited from a more affluent area in the UK. Patterns of co-occurring ACEs may differ in children living in areas of socio-economic disadvantage. Fourth, we did not account for genetic confounding which may explain the association between ACEs and mental health. Our unadjusted and adjusted effect estimates were largely similar, suggesting that the covariates included in the models explained only a small portion of the association. Previous research accounting for genetic confounding has shown that part of the association between ACEs and mental health is explained by genetic risk [56]. Fifth, we did not examine the co-occurrence of ACEs by sex and subsequent mental health outcomes. Previous research has shown that males and females experience different patterns of ACEs. Females are more likely to experience a wide range of co-occurring ACEs than males (four latent classes identified for females vs two for males), such as “High adversity”, “Abuse and neglect”, “Dysfunctional home”, “Low adversity”, versus “Mixed adversity” and “Low adversity” among males [60]. Despite this, patterns of co-occurring ACEs in both males and females were associated with depression in adulthood when compared to the “Low adversity” latent class. Finally, we compared self-harm and depression outcomes among each of the latent classes using the Low ACEs class as the reference group. However, we did not examine whether these outcomes differed between other latent classes by changing the reference group. This limits our understanding of distinctions in how different ACE patterns relate to outcomes and prevents a detailed comparison of risks between specific ACE classes. Future analyses could explore whether certain ACE patterns carry uniquely higher risks relative to others, beyond comparisons to the Low ACEs group.

### Implications

Our findings underscore the importance of considering the patterning of ACEs in childhood and adolescence, in the short and long-term risk of self-harm and depression. Previous research has accounted for the co-occurrence of ACEs by summing up the total number of ACEs experienced and using this ACE score to predict mental health outcomes [26]. However, our results show different patterns of ACEs are differentially associated with mental health. Future research should consider alternative methods of operationalising ACEs beyond the ACE score approaches. These methods should account for their co-occurrence patterning, consider their developmental timing and chronicity, using high quality prospective data.

Prevention of self-harm and depression during adolescence and early adulthood requires primary prevention of ACEs, and early intervention following exposure to mitigate short and long-term impacts. The primary prevention of ACEs through alleviation of financial hardship, promoting societal norms that support healthy relationships, universal intervention for maternal mental illness during pregnancy, and parenting interventions to reduce risk of maltreatment is promising [61–64]. Early childhood abuse and parental mental health problems emerged as a high-risk group for self-harm, depression, and co-occurring self-harm and depression in adolescence, and depression in adulthood. Ongoing multicomponent interventions and multidisciplinary support for children experiencing abuse, and parents with mental illness, during the early childhood period is needed. Early intervention to support parents with mental illness to prevent mental health problems from accruing over time may reduce the long-term risk of mental health problems in children. By distinguishing between different ACE patterns, our study suggests that interventions should address multiple co-occurring ACEs simultaneously and be tailored to the specific developmental trajectories of individuals exposed to these patterns, rather than applying a one-size-fits-all approach to ACE exposure.

## Conclusion

This study found ACEs co-occurring throughout childhood and adolescence are associated with a higher risk of self-harm in adolescence and depression both in adolescence and early adulthood. Additionally, the class most strongly associated with self-harm in early adulthood is Persistent parental mental health problems. Those experiencing Early childhood abuse and parental mental health problems emerged as a high-risk group for self-harm, depression, and co-occurring self-harm and depression in adolescence, and depression in adulthood. Ongoing multicomponent interventions and multidisciplinary support for children experiencing abuse, and parents with mental illness, during this crucial period is needed. These preventive efforts, in combination with timely intervention following exposure, may also reduce the risk of self-harm and depression. Persistent parental mental health problems was most strongly associated with self-harm with co-occurring depression in adulthood. Prevention of parental mental illness from accruing over time may reduce the long-term risk of self-harm and depression.

## Electronic supplementary material

Below is the link to the electronic supplementary material.

Supplementary Material 1

## Data Availability

Informed consent obtained from ALSPAC participants does not allow for the data to be made freely available through any third party maintained public repository. Data used for this article can be made available on request to the ALSPAC Executive. The ALSPAC data management plan describes in detail the policy regarding data sharing, which is through a system of managed open access. Full instructions for applying for data access can be found here: http://www.bristol.ac.uk/alspac/researchers/access/.
