# Supplementary material for: The association between longitudinal patterns of adverse childhood experiences, and self-harm and depression in adolescence and early adulthood: findings from the Avon longitudinal study of parents and children
Source: Eur Child Adolesc Psychiatry. 2025 Jun 6;34(11):3587–99. doi: 10.1007/s00787-025-02781-y (PMC12647182; doi:10.1007/s00787-025-02781-y)
Supplement: Supplementary file 1 — Supplementary Material 1 [file 787_2025_2781_MOESM1_ESM.docx]

**Supplementary material**

**The association between longitudinal patterns of adverse childhood experiences, and self-harm and depression in adolescence and early adulthood: Findings from the Avon Longitudinal Study of Parents and Children**

Bushra Farooq, MSc. Centre for Academic Mental Health, Population Health Sciences, University of Bristol Medical School, Bristol, UK. ORCID: [0000-0002-6435-0881](https://orcid.org/0000-0002-6435-0881)

Abigail E. Russell, PhD. Children and Young People’s Mental Health Research Collaboration, University of Exeter Medical School, Exeter, UK

Kate Allen, PhD. Children and Young People’s Mental Health Research Collaboration, University of Exeter Medical School, Exeter, UK

Laura D. Howe, PhD. MRC Integrative Epidemiology Unit, Population Health Sciences, University of Bristol Medical School, Bristol, UK.

Becky Mars, PhD. Centre for Academic Mental Health, Population Health Sciences, University of Bristol Medical School, National Institute for Health and Care Research, Biomedical Research Centre, Bristol, UK. ORCID: 0000-0002-8132-6920

Corresponding author: Bushra Farooq, [bushra.farooq@bristol.ac.uk](mailto:bushra.farooq@bristol.ac.uk)

**Contents**

| Material S1 | The Avon Longitudinal Study of Parents and Children (ALSPAC) population |
| --- | --- |
| Table S1 | Adverse childhood experiences (ACEs) study definitions |
| Material S2 | Measures of ACEs |
| Material S3 | Measures of self-harm |
| Material S4 | Latent Class Analysis |
| Table S2 | Latent Class model fit indices |
| Table S3 | Probability of each type of ACE for each latent class |
| Table S4 | Prevalence of ACEs by developmental period |
| Table S5 | Comparison of available-cases, proportion of missing data and variables imputed and the multiply imputed sample |
| Table S6 | Comparison of characteristics by different outcomes |
| Table S7 | Adjusted logistic regression models for the association between latent classes and self-harm and depression in adolescent and early adulthood |

**Material S1: The Avon Longitudinal Study of Parents and Children (ALSPAC) study population**

Women resident in Avon, UK, were invited to take part in the study through media campaigns and antenatal services (Boyd, Golding et al. 2013, Fraser, Macdonald-Wallis et al. 2013). The initial number of pregnancies enrolled was 14,541. Of these initial pregnancies, there was a total of 14,676 foetuses, resulting in 14,062 live births and 13,988 children who were alive at 1 year of age (Boyd, Golding et al. 2013, Fraser, Macdonald-Wallis et al. 2013). When the oldest children were approximately 7 years of age, an attempt was made to bolster the initial sample with eligible cases who had failed to join the study originally. As a result, when considering variables collected from the age of seven onwards (and potentially abstracted from obstetric notes) there are data available for more than the 14,541 pregnancies mentioned above: The number of new pregnancies not in the initial sample (known as Phase I enrolment) that are currently represented in the released data and reflecting enrolment status at the age of 24 is 906, resulting in an additional 913 children being enrolled (456, 262 and 195 recruited during Phases II, III and IV respectively). The phases of enrolment are described in more detail in the cohort profile paper and its update (Boyd, Golding et al. 2013, Fraser, Macdonald-Wallis et al. 2013). The total sample size for analyses using any data collected after the age of seven is therefore 15,447 pregnancies, resulting in 15,658 foetuses. Of these 14,901 children were alive at 1 year of age. Of the original 14,541 initial pregnancies, 338 were from a woman who had already enrolled with a previous pregnancy, meaning 14,203 unique mothers were initially enrolled in the study. As a result of the additional phases of recruitment, a further 630 women who did not enrol originally have provided data since their child was 7 years of age. This provides a total of 14,833 unique women (G0 mothers) enrolled in ALSPAC as of September 2021. G0 partners were invited to complete questionnaires by the mothers at the start of the study and they were not formally enrolled at that time. 12,113 G0 partners have been in contact with the study by providing data and/or formally enrolling when this started in 2010. 3,807 G0 partners are currently enrolled (Northstone, Ben Shlomo et al. 2023). The study website contains details of all the data that is available through a fully searchable data dictionary and variable search tool (<http://www.bristol.ac.uk/alspac/researchers/our-data/>). Data were collected via research clinics and postal questionnaires completed by mothers, their partners, and the cohort children themselves (Boyd, Golding et al. 2013, Fraser, Macdonald-Wallis et al. 2013). Study data were collected and managed using REDCap electronic data capture tools hosted at the University of Bristol. REDCap (Research Electronic Data Capture) is a secure, web-based software platform designed to support data capture for research studies (Harris, Taylor et al. 2009).

**Table S1: Adverse childhood experience (ACE) study definitions**

|  | Definition | Informant | Number of questionnaire items | Retrospective questionnaire items |
| --- | --- | --- | --- | --- |
| Physical abuse | Partner/mother was physically cruel to child.  Adult in family pushed, grabbed, shoved/smacked to discipline child. People in child's family hit them so hard that it left them with bruises or marks. | Mother, partner | 36 | 8 |
| Sexual abuse | When growing up someone sexually abused child.  Touched in a sexual way by adult or older child, or was forced to touch adult or older child in a sexual way. Adult or older child forced, or attempted to force, child into any sexual activity by threatening or holding child down or hurting child in some way. | Mother, Child | 7 | 2 |
| Emotional abuse | Partner/mother emotionally cruel to child. Adult in family shouted/ said hurtful or insulting things. | Mother, partner | 37 | 8 |
| Emotional neglect | Carer knows who friends are.  Carer asks/starts conversation about free time/ what happened at school.  Carer takes time to listen when teenager talks about what happened in free time.  Discuss problems with anyone in their family.  Parent/carer talked about child’s experiences at school/ friends/ things that are troubling. Child feels left out of things.  Understood by parents.  When growing up there was someone to take respondent to the doctor if needed.  Someone in family made child feel important or special.  Carer knows what child does with other children. | Mother, Child | 19 | 0 |
| Bullying | Personal belongings stolen, threatened/blackmailed, hit/beaten up.  Do something didn't want to, told lies about child.  Friends tried to get teenager to do things didn’t want to / told lies about teenager.  Young person has been directly/relationally bullied. Child has been bullied.  Upset by name calling/exclusion from groups or bullying.  Someone threatened/blackmailed teenager. | Child | 17 | 0 |
| Parents violent towards each other | Physically cruel.  Kicked, bitten or hit each other, twisted arm, throw body, beaten each other up, choke or strangle each other.  Threatened or used knife or other weapon on each other. | Mother, partner | 46 | 4 |
| Household substance use | Smoked cannabis.  Hard drug use or addiction (crack, heroin, amphetamine, opiate, cocaine, methadone, meth).  Alcoholism/drink problem. Alcohol Use Disorders Identification Test (AUDIT). | Mother, partner | 54 | 0 |
| Parental mental health problems | Parent hurt themselves on purpose or attempted suicide.  Taken medication for depression or anxiety.  Edinburgh Postnatal Depression Scale (EPDS). Diagnosis of schizophrenia, bulimia, anorexia nervosa.  Hospital admission for psychiatric or mental health problems. | Mother, partner, child | 70 | 4 |
| Parent convicted | Court conviction/convicted of an offense. | Mother, partner | 23 | 4 |
| Parental separation | Separated/divorced. | Mother, partner | 39 | 9 |

**Material S2: Measures of ACEs**

There were 348 questionnaire items that measured exposure to ten types of ACEs of interest in this study, covering the exposure period between birth and 16 years. Data on the following eight ACEs were available for all three developmental periods: physical abuse, emotional abuse, sexual abuse, domestic violence, substance abuse, parental mental health problems, parent conviction, and divorce/separation. Additionally, emotional neglect and bullying were only measured during mid-childhood and adolescence. This resulted in 28 binary variables indicating exposure to ten types of ACEs occurring in early childhood, mid-childhood, and adolescence. Data were reported by mothers and their partners when children were 0-8 years, and children themselves and the mothers after the age of 8 years. The table below shows the time point of data collection for each ACE.

|  | Early childhood | Mid-childhood | Adolescence |
| --- | --- | --- | --- |
| Physical abuse | 8 months, 1, 2, 3, and 5 years | 9, and 11 years^*^ | 11 years |
| Emotional abuse | 8 weeks, 8 months, 1, 2, 3, and 5 years | 9, and 11 years^*^ | 11 years |
| Emotional neglect | - | 8, and 9 years | 12, 13, 15, and 16 years |
| Sexual abuse | 1, 2, 3, and 5 years | 9 years | 22 years^*^ |
| Bullying | - | 8, 8.5, and 10 years | 12, 15, and 16 years |
| Domestic violence | 8 months, 1, 2, 3, and 5 years | 8, 9^*^, and 11^*^ years | 11 years |
| Parental mental health problems | 8 weeks, 8 months, 1, 2, 3, and 5 years | 8, 9^*^, 10, and 11^*^ years | 11, 12, and 16 years |
| Substance abuse | 8 weeks, 8 months, 1, 2, 3, and 5 years | 8, and 9 years | 11 years |
| Parent conviction | 8 weeks, 8 months 1, 2, 3, and 5 years | 9, and 11 years^*^ | 11, and 12 years |
| Separation/divorce | 8 months, 1, 2, 3, and 5 years | 9, and 11 years^*^ | 11 years |
| ^*^Retrospective measure | | | |

**Material S3: Measures of self-harm**

At age 16 self-harm was assessed in the ‘Life of a 16+ Teenager’ postal questionnaire. Cohort members were asked “*Have you ever hurt yourself in any way (e.g., by taking an overdose of pills, or by cutting yourself)?*”, followed by “*When was the last time you hurt yourself on purpose?*”, with the following three response categories: “*In the last week/More than a week ago but in the last year/More than a year ago*”. A binary variable was created to indicate past-year self-harm at age 16, responses of “yes” in relation to life-time self-harm, and “*In the last week/More than a week ago but in the last year”* in response to past-year self-harm were coded as *“yes”,* and responses of *“no”* for lifetime self-harm*,* and *“More than a year ago”* in response to past-year self-harm were coded as *“no”.* At age 24 years, this measure was taken from the “Life at 24+” postal questionnaire. The following questions were used to derive a dichotomous past-year self-harm variable: “*Have you ever hurt yourself on purpose in any way (e.g. by taking an overdose of pills or by cutting yourself)?*” and “*If yes, how many times have you done this in the last year?*” , cohort children responded with “*None/Once/2-5 times/6-10 times/More than 10 times*”. Responses of “*yes*” to lifetime self-harm and “*Once/2-5 times/6-10 times/More than 10 times*” in response to the question on frequency in the last year were coded as “*yes*”, responses of “*no*” in response to lifetime self-harm and “*none*” in response to frequency in the past-year were coded as “*no*”.

**Material S4: Latent Class Analysis**

We examined the longitudinal co-occurrence patterns of ACEs using LCA in Mplus, with 28 separate indicators of each ACE (Hagenaars, McCutcheon et al. 2002, Farooq, Allen et al. 2024). Classes were identified using the robust maximum likelihood estimator (Nylund-Gibson and Choi 2018). Over half of the sample had data on more than 50% of the 28 ACE variables, Full Information Maximum likelihood (FIML) was used to handle missing data in the ACE indicators. To determine the optimal class solution we first estimated a two-class model and gradually increased the number of classes up to six. We considered a number of model fit indices when selecting the optimal model: Akaike’s Information Criterion (AIC), Bayesian Information Criterion (BIC), Sample Size Adjusted Bayesian Information Criterion (SSABIC), and the Lo-Mendell-Rubin adjusted likelihood ratio test (LMR LRT). We also considered the interpretability, conceptual meaningfulness, and plausibility of the class solution, class size, and whether the solution was parsimonious (Nylund-Gibson and Choi 2018). We examined the entropy and the average posterior probability (AvePP) to assess classification quality (Weller, Bowen et al. 2020).

**Table S2: Latent Class model fit indices**

|  | Two-class model | Three-class model | Four-class model | Five-class model | Six-class model |
| --- | --- | --- | --- | --- | --- |
| Akaike information criterion (AIC) | 83955·572 | 83369·290 | 82990·923 | 82785·815 | 82662·695 |
| Bayesian information criterion (BIC) | 84359·655 | 83978·960 | 83806·180 | 83806·659 | 83889·125 |
| Sample-size adjusted BIC (SSABIC) | 84178·519 | 83705·667 | 83440·730 | 83349·051 | 83339·360 |
| Lo-Mendell-Rubin adjusted likelihood ratio test (LMR LRT) | 4346·779, p<0·0001 | 641·847, p<0·0001 | 434·717, p=0·0001 | 262·113, p=0·0001 | 180·436, p=0·13 |
| Entropy | 0·661 | 0·672 | 0·695 | 0·708 | 0·722 |

**Table S3: Probability of each type of ACE for each latent class**

|  | Low ACEs (n=6,380) | Mid-childhood and adolescence ACEs (n=230) | Early childhood abuse and parental mental health problems (n=445) | Persistent parental mental health problems (n=861) | Early and mid-childhood household disharmony (n=943) |
| --- | --- | --- | --- | --- | --- |
| Physical abuse (birth-5 years) | 0.009 | 0.022 | 0.547 | 0.026 | 0.066 |
| Sexual abuse (birth-5 years) | 0 | 0.009 | 0.001 | 0.004 | 0.008 |
| Emotional abuse (birth-5 years) | 0.041 | 0.092 | 0.856 | 0.12 | 0.275 |
| Domestic violence (birth-5 years) | 0.009 | 0.124 | 0.373 | 0.03 | 0.343 |
| Substance abuse (birth-5 years) | 0.029 | 0.061 | 0.115 | 0.073 | 0.179 |
| Parental mental health (birth-5 years) | 0.179 | 0.308 | 0.699 | 0.65 | 0.602 |
| Parent conviction (birth-5 years) | 0.03 | 0.036 | 0.113 | 0.042 | 0.134 |
| Separation/divorce (birth-5 years) | 0.059 | 0.178 | 0.317 | 0.093 | 0.636 |
| Physical abuse (age 6-10 years) | 0 | 0.137 | 0.127 | 0.001 | 0 |
| Emotional abuse (age 6-10 years) | 0.007 | 0.55 | 0.362 | 0.026 | 0.024 |
| Emotional neglect (age 6-10 years) | 0.022 | 0.045 | 0.04 | 0.028 | 0.058 |
| Bullying (age 6-10 years) | 0.112 | 0.176 | 0.148 | 0.144 | 0.211 |
| Domestic violence (age 6-10 years) | 0.077 | 0.441 | 0.491 | 0.17 | 0.343 |
| Substance abuse (age 6-10 years) | 0.012 | 0.019 | 0.079 | 0.036 | 0.094 |
| Parental mental health (age 6-10 years) | 0.047 | 0.263 | 0.37 | 0.735 | 0.21 |
| Parent conviction (age 6-10 years) | 0.007 | 0.037 | 0.034 | 0.015 | 0.018 |
| Separation/divorce (age 6-10 years) | 0.03 | 0.595 | 0.17 | 0.088 | 0.311 |
| Sexual abuse (age 6-10 years) | 0 | 0 | 0 | 0 | 0.005 |
| Physical abuse (age 11-16 years) | 0 | 0.043 | 0.021 | 0 | 0 |
| Emotional abuse (age 11-16 years) | 0.001 | 0.194 | 0.128 | 0.004 | 0.004 |
| Emotional neglect (age 11-16 years) | 0.146 | 0.245 | 0.135 | 0.134 | 0.186 |
| Bullying (age 11-16 years) | 0.099 | 0.107 | 0.164 | 0.132 | 0.096 |
| Domestic violence (age 11-16 years) | 0 | 0.081 | 0.037 | 0.007 | 0.005 |
| Substance abuse (age 11-16 years) | 0 | 0 | 0 | 0.005 | 0.003 |
| Parental mental health (age 11-16 years) | 0.026 | 0.191 | 0.241 | 0.427 | 0.086 |
| Parent conviction (age 11-16 years) | 0.003 | 0.014 | 0.016 | 0.002 | 0.01 |
| Separation/divorce (age 11-16 years) | 0.006 | 0.271 | 0.037 | 0.02 | 0.028 |
| Sexual abuse (age 11-17 years) | 0.05 | 0.065 | 0.098 | 0.086 | 0.067 |

**Table S4: Prevalence of ACEs by developmental period**

|  | Early childhood n (%) | Mid-childhood n (%) | Adolescence n (%) |
| --- | --- | --- | --- |
| Physical abuse | 404 (5·2) | 88 (1·3) | 24 (0·3) |
| Emotional abuse | 883 (13·1) | 359 (5·3) | 142 (1·6) |
| Emotional neglect^^^ | - | 235 (2·9) | 782 (15·1) |
| Bullying^^^ | - | 912 (13·2) | 946 (10·7) |
| Domestic violence | 620 (7·9) | 906 (15·1) | 56 (0·6) |
| Substance abuse^*^ | 449 (5·9) | 177 (2·8) | <5 (0·1) |
| Parental mental health | 2,563 (32·3) | 1,259 (17·8) | 876 (9·9) |
| Parent conviction | 395 (5·0) | 82 (1·2) | 45 (0·5) |
| Separation/divorce | 1,208 (15·6) | 570 (9·2) | 190 (2·1) |
| Sexual abuse | 18 (0·2) | 6 (0·1) | 199 (5·9) |
| Denominator differs for each adverse childhood experience  ^*^Numbers are not reported where cell count is less than five (this may include zero)  ^^^Not measured in early childhood | | | |

**Table S5: Comparison of available-cases, proportion of missing data and variables imputed and the multiply imputed sample**

|  | N missing values (% imputed) | Available cases (n=3,363-8,859) (%)^*^ | Pooled proportions from multiply imputed data (n=8,859)^^^ |
| --- | --- | --- | --- |
| *Outcomes* | | | |
| Self-harm (adolescence) | 4,356 (49.2) | 487 (10.8) | 11.8 |
| Self-harm (early adulthood) | 5,285 (59.7) | 274 (7.7) | 9.7 |
| Depression (adolescence)^a^ | 4,412 (49.8) | 665 (15.0) | 16.0 |
| Depression (early adulthood)^a^ | 5,496 (62.0) | 691 (20.6) | 22.2 |
| *Characteristics* | | | |
| Male | N/A | 4,495 (50.7) | 50.7 |
| Ethnicity of child | 393 (4.4) | - | - |
| Non-white | - | 330 (3.9) | 4.0 |
| White | - | 8,136 (96.1) | 96.0 |
| Household social class | 494 (5.6) | - | - |
| Professional | - | 887 (10.6) | 10.4 |
| Managerial and technical | - | 3,404 (40.7) | 40.2 |
| Skilled non-manual | - | 2,548 (30.5) | 30.5 |
| Skilled manual | - | 935 (11.2) | 11.4 |
| Partly skilled or unskilled | - | 591 (7.1) | 7.4 |
| Housing | 225 (2.5) | - | - |
| Owned/mortgaged | - | 7,016 (81.3) | 81.1 |
| Rented | - | 1,379 (16.0) | 16.1 |
| Other | - | 239 (2.8) | 2.8 |
| Mother’s qualifications^b^ | 241 (2.7) | - | - |
| CSE | - | 1,264 (14.7) | 14.9 |
| Vocational | - | 776 (9.0) | 9.1 |
| O level | - | 3,072 (35.7) | 35.7 |
| A level | - | 2,189 (25.4) | 25.3 |
| Degree | - | 1,317 (15.3) | 15.1 |
| Mother’s age at delivery | N/A | - | - |
| Under 20 years | - | 201 (2.3) | 2.3 |
| 20-29 years | - | 4,817 (54.4) | 54.4 |
| 30 plus years | - | 3,841 (43.4) | 43.4 |
| Parity (mean) | 267 (3.0) | 8,592 (0.79) | 0.80 |
| ^*^ Using all the available data for each variable separately, denominator may differ  ^^^Only pooled proportions from multiply imputed data are presented as numbers vary in each imputed dataset  ^a^Measured using the Short Mood and Feelings Questionnaire (SMFQ)  ^b^Certificate of Secondary Education (CSE) consists of grades 1 to 5, grade 1 is equivalent to pass at Ordinary level (O level). CSEs and O levels merged into General Certificate of Secondary Education (GCSE) qualifications. Advanced level (A level) qualifications are undertaken by students aged 16 and above, taken over two years leading to qualifications for entrance into higher education. Degree refers to university qualifications.  N/A refers to no missing data | | | |

**Table S6: Comparison of characteristics by different outcomes**

|  | Adolescent self-harm | Adolescent depression | No self-harm and depression in adolescence | Early adulthood self-harm | Early adulthood depression | No self-harm and depression in early adulthood |
| --- | --- | --- | --- | --- | --- | --- |
| *Characteristics* | | | | | | |
| Male | 34.2 | 36.9 | 54.9 | 43.8 | 45.7 | 52.6 |
| Female | 65.8 | 63.1 | 45.1 | 56.2 | 54.3 | 47.4 |
| Ethnicity of child | | | | | | |
| Non-white | 4.2 | 4.6 | 3.9 | 4.1 | 4.9 | 3.8 |
| White | 95.8 | 95.4 | 96.1 | 95.9 | 95.1 | 96.2 |
| Household social class | | | | | | |
| Professional | 8.5 | 7.6 | 11.0 | 7.7 | 8.0 | 11.2 |
| Managerial and technical | 37.5 | 40.7 | 40.6 | 35.7 | 35.5 | 41.8 |
| Skilled non-manual | 29.7 | 28.4 | 30.7 | 31.0 | 31.2 | 30.3 |
| Skilled manual | 14.9 | 13.5 | 10.8 | 12.5 | 16.8 | 10.0 |
| Partly skilled or unskilled | 9.4 | 9.9 | 6.9 | 13.2 | 8.6 | 6.6 |
| Housing | | | | | | |
| Owned/mortgaged | 73.5 | 78.3 | 82.2 | 73.6 | 75.3 | 83.2 |
| Rented | 22.1 | 18.7 | 15.2 | 23.4 | 21.3 | 14.2 |
| Other | 4.4 | 2.9 | 2.6 | 3.1 | 3.4 | 2.6 |
| Mother’s qualifications^b^ | | | | | | |
| CSE | 15.0 | 16.4 | 14.6 | 21.5 | 17.5 | 13.7 |
| Vocational | 7.0 | 10.7 | 9.0 | 6.6 | 9.7 | 9.1 |
| O level | 39.4 | 39.7 | 34.8 | 40.0 | 36.6 | 35.3 |
| A level | 22.5 | 22.0 | 26.0 | 20.0 | 24.9 | 25.6 |
| Degree | 16.0 | 11.2 | 15.6 | 11.9 | 11.2 | 16.3 |
| Mother’s age at delivery | | | | | | |
| Under 20 years | 4.1 | 3.2 | 1.9 | 4.4 | 4.1 | 1.7 |
| 20-29 years | 58.6 | 56.0 | 53.8 | 54.5 | 55.0 | 54.2 |
| 30 plus years | 37.4 | 40.8 | 44.3 | 41.1 | 40.9 | 44.1 |
| Parity (mean) | 0.81 | 0.82 | 0.79 | 0.91 | 0.77 | 0.79 |
| ^a^ Measured using the Short Mood and Feelings Questionnaire (SMFQ)  ^b^Certificate of Secondary Education (CSE) consists of grades 1 to 5, grade 1 is equivalent to pass at Ordinary level (O level). CSEs and O levels merged into General Certificate of Secondary Education (GCSE) qualifications. Advanced level (A level) qualifications are undertaken by students aged 16 and above, taken over two years leading to qualifications for entrance into higher education. Degree refers to university qualifications. | | | | | | |

**Table S7: Adjusted logistic regression models for the association between latent classes and self-harm and depression in adolescent and early adulthood.**

|  | Self-harm  OR (95% CI) | Depression  OR (95% CI) | Co-occurring self-harm and depression  OR (95% CI) | Self-harm  OR (95% CI) | Depression  OR (95% CI) | Co-occurring self-harm and depression  OR (95% CI) |
| --- | --- | --- | --- | --- | --- | --- |
|  | Adolescence (n=8,859) | | | Early adulthood (n=8,859) | | |
| Low ACEs (reference group; n=6,380) | | | | | | |
| Mid-childhood and adolescence ACEs (n=230) | 1.85 (1.16-2.93) | 1.19 (0.71-1.97) | 1.68 (0.86-3.29) | 1.18 (0.67-2.08) | 1.68 (1.13-2.50) | 1.66 (0.85-3.24) |
| Early childhood abuse and parental mental health problems (n=445) | 1.86 (1.34-2.58) | 2.81 (2.12-3.72) | 2.43 (1.62-3.64) | 0.98 (0.64-1.50) | 2.18 (1.66-2.85) | 0.99 (0.54-1.82) |
| Persistent parental mental health problems (n=861) | 1.33 (0.98-1.80) | 1.43 (1.08-1.87) | 1.32 (0.86-2.04) | 1.48 (1.10-2.01) | 1.83 (1.45-2.31) | 1.98 (1.36-2.88) |
| Early and mid-childhood household disharmony (n=943) | 1.56 (1.17-2.07) | 1.35 (1.01-1.80) | 1.67 (1.12-2.49) | 1.08 (0.76-1.53) | 1.37 (1.07-1.77) | 1.21 (0.76-1.93) |
| Models adjusted for poverty and sex.  Poverty was defined as difficulties in affording food, heating, and accommodation, and parents becoming homeless at any time during the pregnancy.  OR = odds ratio  CI = confidence interval  ACEs = adverse childhood experiences | | | | | | |

Boyd, A., J. Golding, J. Macleod, D. A. Lawlor, A. Fraser, J. Henderson, L. Molloy, A. Ness, S. Ring and G. D. Smith (2013). "Cohort Profile: the 'children of the 90s'--the index offspring of the Avon Longitudinal Study of Parents and Children." International Journal of Epidemiology **42**(1): 111-127.

Farooq, B., K. Allen, A. E. Russell, L. D. Howe and B. Mars (2024). "The association between poverty and longitudinal patterns of adverse childhood experiences across childhood and adolescence: Findings from a prospective population-based cohort study in the UK." Child Abuse & Neglect **156**.

Fraser, A., C. Macdonald-Wallis, K. Tilling, A. Boyd, J. Golding, G. Davey Smith, J. Henderson, J. Macleod, L. Molloy, A. Ness, S. Ring, S. M. Nelson and D. A. Lawlor (2013). "Cohort Profile: the Avon Longitudinal Study of Parents and Children: ALSPAC mothers cohort." International Journal of Epidemiology **42**(1): 97-110.

Hagenaars, J. A., A. L. McCutcheon and ProQuest (2002). Applied latent class analysis. Cambridge ; New York, Cambridge University Press.

Harris, P. A., R. Taylor, R. Thielke, J. Payne, N. Gonzalez and J. G. Conde (2009). "Research electronic data capture (REDCap)--a metadata-driven methodology and workflow process for providing translational research informatics support." Journal of Biomedical Informatics **42**(2): 377-381.

Northstone, K., Y. Ben Shlomo, A. Teyhan, A. Hill, A. Groom, M. Mumme, N. Timpson and J. Golding (2023). "The Avon Longitudinal Study of Parents and children ALSPAC G0 Partners: A cohort profile [version 1; peer review: 1 approved with reservations]." Wellcome Open Research **8**(37).

Nylund-Gibson, K. and A. Y. Choi (2018). "Ten frequently asked questions about latent class analysis." Translational Issues in Psychological Science **4**(4): 440-461.

Weller, B. E., N. K. Bowen and S. J. Faubert (2020). "Latent Class Analysis: A Guide to Best Practice." Journal of Black Psychology **46**(4): 287-311.
